# Safety and feasibility of one-stage neonatal approach for short-segment Hirschsprung’s disease

**DOI:** 10.1371/journal.pone.0341212

**Published:** 2026-01-16

**Authors:** Quynh Anh Tran, Hien Duy Pham, Dung Boi Ly, Minh Quang Ngo, Nhung Thi Nguyen, Liem Thanh Nguyen, Quang Thanh Nguyen

**Affiliations:** 1 Department of Surgery, The National Hospital of Pediatrics, Dong Da, Hanoi, Vietnam; 2 College of Health Sciences, VinUniversity, Gia Lam, Hanoi, Vietnam; Macau University of Science and Technology, HONG KONG

## Abstract

**Background:**

Early definitive surgery for Hirschsprung disease (HD) in neonates is increasingly adopted to reduce preoperative morbidity and preserve long term bowel function. However, comparative data across minimally invasive approaches in neonates with short segment disease remain limited. This study compared outcomes of single incision laparoscopic assisted endorectal pull through (SILEP), conventional laparoscopic assisted endorectal pull through (CLEP), and complete transanal endorectal pull through (TERPT) for rectosigmoid HD.

**Methods:**

We conducted a retrospective cohort study of 55 neonates who underwent one stage definitive surgery before 28 days of age at a high volume center between January 2019 and December 2021. The primary outcome was long term bowel function assessed using the Rintala Bowel Function Score (BFS) after a minimum of 4 years of follow up. Secondary outcomes included operative parameters, postoperative complications (Clavien Dindo classification), and cosmetic outcomes using the Manchester Scar Scale (MSS) in the laparoscopic groups.

**Results:**

All patients successfully underwent surgery at a mean age of 22.4 ± 4.3 days. Operative time differed across approaches and was shorter for SILEP (53.8 ± 11.9 minutes) and TERPT (52.1 ± 18.3 minutes) than for CLEP (70.2 ± 22.5 minutes, p = 0.036). At follow up (mean 54.0 ± 7.7 months), the overall BFS was 17.5 ± 2.5 with no significant differences among groups (p = 0.32). MSS was numerically lower for SILEP than for CLEP (6.2 ± 1.1 vs 6.8 ± 1.9, p = 0.53). Complications were infrequent, with 14 minor and 7 major events, and there was no mortality or Clavien Dindo grade IV or V morbidity.

**Conclusion:**

SILEP, CLEP, and TERPT are feasible one stage options for neonates with rectosigmoid HD, with comparable long term bowel function and low rates of major complications. SILEP and TERPT were associated with shorter operative times, and SILEP showed a trend toward improved cosmetic scores compared with CLEP. These findings support an individualized approach to technique selection based on intraoperative requirements and institutional expertise.

## Introduction

Hirschsprung’s disease (HD) is a congenital disorder of intestinal motility caused by the absence of ganglion cells in the distal bowel, leading to chronic functional obstruction [1]. Definitive treatment requires resection of the aganglionic segment and pull-through of normally innervated bowel. Over recent decades, surgical management has shifted towards minimally invasive approaches, particularly in neonates, where early intervention may prevent colonic distension and irreversible neuromuscular changes, thereby improving long-term bowel function [1].

The Transanal Endorectal Pull-Through (TERPT), introduced in the late 1990s, rapidly gained acceptance due to its scarless access, reduced operative trauma, and favorable outcomes in neonates, where soft tissue planes and shorter bowel length facilitate dissection [2,3]. Despite these advantages, concerns persist regarding prolonged anal retraction, sphincter overstretching, and their potential impact on continence and anastomotic healing. To address these limitations, laparoscopic-assisted techniques were introduced, including Conventional Laparoscopic Endorectal Pull-through (CLEP) and Single-Incision Laparoscopic Endorectal Pull-through (SILEP). Both allow improved visualization of the transition zone, tension-free mobilization of the colon, and potentially lower risks of residual aganglionosis [4–6]. Among them, SILEP is particularly appealing for minimizing abdominal trauma and optimizing cosmetic outcomes, although its technical demands and specialized equipment have limited widespread adoption [5,6].

While several meta-analyses have compared TERPT and laparoscopic-assisted procedures, they often group SILEP and CLEP together [4,7,8]. Thomson et al. [7] found TERPT to have shorter operative times with comparable functional outcomes. On the other hand, Celtik et al. [8] reported lower rates of constipation and enterocolitis after TERPT compared with CLEP, though other complications were similar. In contrast, van de Ven et al. [4] described higher rates of colonic torsion with TERPT, underlining the benefit of laparoscopic support for intraperitoneal visualization. The timing of surgery also remains debated. Some recommend delaying definitive repair to allow colonic decompression and better localization of the transition zone [9–11], whereas growing evidence supports early neonatal intervention for reducing Hirschsprung-associated enterocolitis (HAEC) and preserving bowel motility [1,12].

At our institution, two recent studies have reported encouraging outcomes with SILEP in neonates [13,14]; however, they did not include comparisons with other commonly used techniques such as TERPT or CLEP. Building on this experience, the present study evaluates neonatal outcomes across all three approaches. The primary objective was to assess long-term functional results using validated scoring systems, while secondary objectives included complication profiles, operative parameters, and cosmetic outcomes. Conducted at a high-volume referral center, this study provides robust and clinically relevant evidence to guide surgical decision-making and optimize care for this vulnerable patient population.

## Methods

### Ethics approval

This study was approved by the Institutional Review Board of VinUniversity under the oversight of the Ethics Committee in Biomedical Research of Vinmec International General Hospital, Hanoi, Vietnam (Approval No. 147/2023/CN-HĐĐĐ VMEC, dated 17 March 2023). All procedures performed in this study were in accordance with the ethical standards of the institutional research committee and with the 1964 Helsinki declaration and its later amendments. Due to the retrospective and observational nature of the study, the requirement for informed consent was waived by the ethics committee.

### Study design

This retrospective cohort study was conducted at the Department of Pediatric Surgery, Vietnam National Hospital of Pediatrics, evaluating neonates diagnosed with HD who underwent definitive single-stage surgical treatment between January 1, 2019, and December 31, 2021. Study data were accessed for research purposes between January 2024 and September 2024. During this period, only two members of the research team had access to directly identifiable participant information for the purposes of data extraction and verification. After this stage, all data were deidentified and transferred into a template worksheet, and all subsequent data management and analyses were performed using this deidentified dataset. No other personnels had access to information that could identify individual participants during or after data collection. Clinical data were collected and reported in adherence to the Strengthening the Reporting of Observational Studies in Epidemiology (STROBE) guidelines.

### Patient selection

Eligible patients were neonates (≤28 days old at the time of surgery) with a postoperative histopathology which confirmed the diagnosis of HD limited to the rectosigmoid segment. Patients with proximal disease, including left-colon aganglionosis, were excluded to restrict the cohort to short-segment HD and minimize confounding related to the distinct technical demands and prognostic outcomes of long-segment disease. All patients underwent either transanal (TERPT), single-incision (SILEP), or conventional three-trocar (CLEP) approach, and all procedures were performed by a single dedicated surgical team. Patients were excluded if they had any of the following criteria: (1) left-colon or more proximal aganglionosis including total colonic aganglionosis confirmed on histopathology; (2) presence of syndromic conditions such as Down syndrome or major associated congenital anomalies affecting other organ systems; (3) incomplete or missing clinical, surgical, or follow-up data; or (4) patients who underwent staged surgical treatment, including those who had initial diverting stoma or delayed definitive repair.

In this manuscript, we make use of a subset of intraoperative variables from neonates who underwent single incision laparoscopic assisted endorectal pull through (SILEP) that were previously reported in our prospective study focusing exclusively on SILEP medium term outcomes in neonates with Hirschsprung disease [13]. These overlapping intraoperative data are used only as part of a broader comparative analysis between three distinct neonatal approaches SILEP, CLEP, and TERPT, and are integrated with additional perioperative, functional, cosmetic, and complication data that were not included in the earlier article. The two studies address clearly different research questions and objectives, with the prior work evaluating the safety and medium-term results of a single technique, whereas the current study investigates long term functional and cosmetic outcomes across multiple operative strategies to guide surgical decision making in neonatal Hirschsprung surgery.

### Surgical approaches

TERPT was performed entirely via a transanal approach without any laparoscopic assistance, following the Soave endorectal pull-through technique. In the laparoscopic group, mobilization of the colon was performed laparoscopically up to the peritoneal reflection before transanal dissection. The SILEP procedure utilized conventional laparoscopic instruments and followed the technique previously described by Nguyen et al. [6]. The choice of SILEP versus CLEP was based on the surgeon’s judgment after the initial laparoscopic assessment. CLEP was usually selected rather than SILEP in cases of marked bowel distension with limited visualization, a proximal or uncertain transition zone requiring extensive mobilization and multiple leveling biopsies, concern for mesenteric tension or compromised perfusion, significant bowel edema or friability consistent with enterocolitis, and other anatomic or technical factors that reduced exposure or instrument maneuverability with a single-incision approach.

### Follow-up data

The primary outcome was long-term bowel function, assessed using the Rintala Bowel Function Score (BFS) at follow-up for all patients aged four years or older.

Secondary outcomes included postoperative complications, cosmetic appearance, and perioperative variables. Complications were classified according to the Clavien–Dindo system [15], with details recorded for type, severity, timing, and required interventions. They were further categorized as minor (Grade I–II) or major (Grade IIIb and above). Cosmetic outcomes were assessed in patients who underwent SILEP or CLEP using the Manchester Scar Scale (MSS), which evaluates scar color, contour, distortion, texture, and overall appearance on a five-point Likert scale, with lower scores indicating superior results [16]. Additional perioperative variables included operative time, age at surgery, hospital length of stay, and the level of aganglionosis as confirmed by histopathology. These data were compared across groups to examine associations between surgical technique and outcomes.

### Statistical analysis

Statistical analyses were performed using R Statistical Software version 4.3.0 (R Core Team, Vienna, Austria). Categorical variables were summarized as frequencies and % and compared between surgical groups using Fisher exact testing. For contingency tables larger than 2 by 2, or when expected cell counts were small, we used the Fisher Freeman Halton extension of the Fisher exact test for R by C tables. This approach is appropriate for nominal categorical variables without inherent ordering and avoids assumptions required by ordinal or rank based methods. Continuous variables were summarized as means plus standard deviation for approximately normally distributed data, or as medians with interquartile ranges for nonnormal distributions. Comparisons across the three surgical approaches were conducted using one way analysis of variance for approximately normal continuous outcomes, or the Kruskal Wallis rank sum test when distributional assumptions were not met. When clinically relevant, a secondary comparison combined the two laparoscopic assisted approaches (SILEP plus CLEP) and compared them with TERPT using Fisher exact testing for categorical outcomes and either a two sample t test or Wilcoxon rank sum test for continuous outcomes, as appropriate. All tests were two sided, and a p value less than 0.05 was considered statistically significant.

## Results

Table 1 summarizes the demographic and operative characteristics of the study population. Between January 2019 and December 2021, 55 neonates underwent definitive one-stage surgical treatment for HD at our institution. The mean age at surgery was 22.4 ± 4.3 days and differed modestly among groups (p = 0.045). Operative time was significantly shorter in the SILEP and TERPT groups compared with CLEP (p < 0.05).

**Table 1:** Demographic and operative characteristics.

Variable	Overall (n = 55)	SILEP (n = 23)	CLEP (n = 22)	TERPT (n = 10)	p-value*
Gender, n (%)					0.18
Female	8 (15%)	1 (4.3%)	5 (23.0%)	2 (20.0%)	
Male	47 (85%)	22 (95.7%)	17 (77.0%)	8 (80.0%)	
Age at surgery (days)	22.4 ± 4.3	22.4 ± 3.2	21.3 ± 5.3	24.9 ± 3.2	0.045
Operative time (minutes)	60.3 ± 12.5	53.8 ± 11.9	70.2 ± 22.5	52.1 ± 18.3	0.036
Length of hospital stay (days)	5.1 ± 2.0	4.5 ± 1.1	5.6 ± 2.4	5.5 ± 2.1	0.065
Location of aganglionosis, n (%)					0.21
Rectum	33 (60%)	11 (47.8)	13 (59%)	9 (90%)	
Sigmoid	22 (40%)	12 (52.2%)	9 (41%)	1 (10%)	

* Fisher’s exact test; Kruskal-Wallis rank sum test.

Table 2 presents the surgical outcomes following the three techniques. The overall mean follow-up duration was 54.0 ± 7.7 months. Cosmetic outcomes, assessed using the MSS for patients undergoing laparoscopic procedures, yielded an overall mean of 6.5 ± 1.6, with SILEP showing lower scores compared to those of CLEP, indicating more favorable cosmetic results. At final follow-up, all patients were older than four years and underwent bowel function assessment using the BFS. The overall mean Rintala score was 17.5 ± 2.5, reflecting favorable long-term outcomes. Bowel function scores did not differ significantly among SILEP, CLEP, and TERPT (p = 0.32). In an additional comparison of laparoscopic assisted approaches combined (SILEP plus CLEP) versus TERPT, no significant difference was observed (p = 0.8). Complication analysis showed 14 minor (Clavien–Dindo Grade I & II) and 7 major events (Clavien–Dindo Grade IIIb and higher), without significant differences between laparoscopic and transanal groups (p = 0.56 and p = 0.58, respectively). No mortality or severe morbidity occurred in this cohort.

**Table 2:** Surgical outcomes of three techniques for Hirschsprung disease.

Variable	Overall (n = 55)	SILEP (n = 23)	CLEP (n = 22)	TERPT (n = 10)	p-value*
Follow-up time (months)	54.0 ± 7.7	54.1 ± 4.2	53.6 ± 9.3	55.1 ± 10.6	0.76
Rintala’s BFS	17.5 ± 2.5	17.2 ± 2.1	17.7 ± 3.0	17.6 ± 2.3	0.32
Manchester Scar Score	6.5 ± 1.6	6.2 ± 1.1	6.8 ± 1.9	N/A	0.53
**Postoperative Complications (Clavien–Dindo)**					
Grade I & II	14 (25.5%)	7 (30.4%)	6 (27.3%)	1 (10.0%)	0.56
• Anal abscess	1 (1.8%)	1 (4.3%)	0 (0.0%)	0 (0.0%)	
• Enterocolitis	10 (18.2%)	6 (26%)	3 (13.6%)	1 (10.0%)	
• Wound dehiscence	1 (1.8%)	0 (0%)	1 (4.5%)	0 (0.0%)	
• Wound infection	2 (3.6%)	0 (0%)	2 (9.1%)	0 (0.0%)	
Grade IIIb	7 (12.7%)	2 (8.7%)	3 (13.6%)	2 (20.0%)	0.58
• Bowel adhesion	2 (3.6%)	0 (0.0%)	2 (9.1%)	0 (0.0%)	
• Anastomotic stenosis	2 (3.6%)	2 (8.7%)	0 (0.0%)	0 (0.0%)	
• Perianal abscess	1 (1.8%)	0 (0.0%)	0 (0.0%)	1 (10.0%)	
• Recurrence	1 (1.8%)	0 (0.0%)	0 (0.0%)	1 (10.0%)	
• Rectovaginal fistula	1 (1.8%)	0 (0.0%)	1 (4.5%)	0 (0.0%)	
Grade IV-V	0 (0.0%)	0 (0.0%)	0 (0.0%)	0 (0.0%)	

* Fisher’s exact test, Kruskal-Wallis rank sum test.

## Discussion

### Safety and feasibility of neonatal surgery

The optimal timing for surgical intervention in Hirschsprung’s disease (HD) remains a subject of debate. While some guidelines recommend postponing surgery to allow for bowel decompression and better transition zone identification [9,11], there is growing support for early neonatal surgery as both feasible and safe. Those in favor of delayed intervention highlight the physiological immaturity of the neonatal bowel, specifically underdeveloped motility and tissue fragility, which may increase the risk of postoperative complications such as dysmotility, feeding intolerance, and anastomotic stricture or leakage [10,17,18]. These concerns are especially relevant in centers with limited neonatal surgical experience, where accurate identification of the transition zone can be challenging.

However, the risks of delayed surgery are not negligible. Beltman et al. [19] reported that 17% of patients awaiting definitive repair developed significant complications, including bowel perforation. Liu et al. [20] further emphasized this by documenting a 6% perforation rate among neonates awaiting surgery. These findings underscore the importance of timely intervention to avoid potentially life-threatening preoperative deterioration.

In our series, all 55 patients underwent definitive pull-through surgery within the neonatal period (≤28 days), allowing for a focused evaluation of early outcomes. The average age at surgery was 22.4 ± 4.3 days, which compares favorably with previous studies [3,21]. Zhang et al. [21] performed TERPT at a median age of 17 days and reported that 80.2% of children achieved long-term bowel continence (BFS ≥ 18). Similarly, Liu et al. [3] found that 83.8% of neonates who underwent neonatal TERPT maintained BFS ≥ 17 after 7.3 years of follow-up. All patients were over 4 years old at their final follow-up and were assessed using the standardized BFS, enabling meaningful long-term evaluation. The overall mean BFS was 17.5 ± 2.5, with no significant differences among the groups (p = 0.32).

Another key consideration in neonatal HD surgery is the risk of HAEC. Xia et al. [1] demonstrated that early intervention reduced HAEC incidence from 43.4% preoperatively to 20.2% postoperatively. In our cohort, the overall rate of HAEC was 18.2%, suggesting that early surgery may help mitigate enterocolitis risk.

Our data on surgical complications further supports the safety of early neonatal repair. No patients experienced Clavien-Dindo Grade IV or V complications, and there were no mortalities. Minor complications occurred in 14 patients, and major complications in 7, with no significant differences between the laparoscopic (SILEP and CLEP) and transanal groups. Besides, our Grade IIIb complication rate of 12.7% aligns with findings from Roy and Jaffray [22], whose data also reported no significant differences in major outcomes between early and delayed surgery groups.

Additionally, early neonatal surgery may offer anatomical advantages. Neonates typically present with thinner abdominal walls, less colonic dilation, and reduced mesenteric fat, facilitating easier laparoscopic mobilization and endorectal dissection. These technical benefits are reflected in our operative data: the mean operative time for all patients was 60.3 ± 12.5 minutes, with even shorter durations in the SILEP (53.8 ± 11.9 minutes) and TERPT (52.1 ± 18.3 minutes) groups. These findings are consistent with other reports [1,3] highlighting increased efficiency and favorable anatomy in neonates undergoing early repair.

### Choice of operative approaches

Various operative techniques are used to treat HD in neonates, each offering specific advantages. Among laparoscopic approaches, SILEP has gained popularity due to reduced surgical trauma and superior cosmetic outcomes, attributed to a single concealed umbilical incision [23,24]. In our cohort, the average hospital stay for SILEP was 4.5 ± 1.1 days, shorter than that for CLEP, which averaged 5.6 ± 2.4 days. The mean Manchester Scar Score was also better in the SILEP group (6.2 ± 1.1) than in the CLEP group (6.8 ± 1.9), reflecting improved cosmesis. These findings support previously reported benefits of SILEP in terms of faster recovery and enhanced aesthetic results [6,23,24]. Cosmetic outcomes are critical in neonates, as abdominal scars tend to enlarge with growth [23,24].

Although technical challenges such as instrument crowding and reduced triangulation have been noted in SILEP procedures [25,26], multiple studies have demonstrated that SILEP offers surgical efficacy comparable to CLEP [26,27]. Our findings are consistent with this, showing that SILEP had a significantly shorter mean operative time (53.8 ± 11.9 minutes) compared to CLEP (70.2 ± 22.5 minutes), likely reflective of increasing technical proficiency and surgeon experience.

TERPT, meanwhile, continues to be a dependable technique with consistently favorable outcomes. In our study, it had the shortest operative time (52.1 ± 18.3 minutes) and a mean BFS of 17.6 ± 2.3. Postoperative complication rates did not differ significantly between groups, supporting comparable safety profiles. When both laparoscopic approaches (SILEP and CLEP) were grouped and compared with TERPT, no significant differences were observed in long-term bowel function. The mean Rintala score was 17.4 ± 2.7 in the combined laparoscopic group versus 17.6 ± 2.3 in the TERPT group (p = 0.8), indicating similar overall efficacy.

Findings from other studies support the equivalence of these techniques. For example, Ho et al. [28] showed that TERPT had shorter operative times than the laparoscopic-assisted group with similar stooling and complication outcomes. Elrouby et al. [29] found that one-stage transanal and laparoscopic-assisted approaches both yielded effective results, with minor procedural differences. Additionally, Wang et al. [30] and Guerra et al. [2] reported no significant differences in enterocolitis rates or long-term bowel function between total transanal and laparoscopic-assisted techniques.

Robotic-assisted surgery has introduced a new dimension in the treatment of HD, primarily through enhanced precision and visualization. Zhang et al. [31] demonstrated that robotic-assisted pull-throughs in infants younger than three months were as safe as those in older children, with comparable continence and complication profiles. Similarly, Hou et al. [32] reported reduced intraoperative blood loss and equivalent safety in neonates undergoing robotic-assisted Swenson pull-through. At our center, however, robotic surgery has not been adopted in neonates because of limitations inherent to the platform, particularly its multiport configuration and the large size of the instruments [33]. These anatomical constraints have led us to rely exclusively on SILEP, CLEP, and TERPT in the neonatal population. This approach is consistent with broader surgical trends, where robotic platforms are more commonly applied in older infants and children rather than in neonates. Our findings, together with the existing literature, including a recent systematic review and meta-analysis of minimally invasive approach for HD [34], support that SILEP, CLEP, and TERPT are safe and effective operative options in neonates.

### Limitations

This study should be interpreted in light of certain limitations. As a retrospective analysis, allocation of surgical techniques was determined by surgeon preference and intraoperative findings rather than randomization, which may introduce selection bias. Nevertheless, this reflects real-world clinical decision-making in a high-volume center. Although all patients were followed for at least four years, allowing reliable assessment of early functional outcomes, longer follow-up into later childhood and adolescence will be essential to capture continence, social toileting, and quality of life outcomes that evolve over time. The number of patients in the TERPT group was smaller compared with the laparoscopic groups, which may limit the power of subgroup comparisons, although the overall cohort remains one of the largest neonatal series reported to date. Finally, robotic surgery was not included because current platforms require multiport access and relatively large instruments unsuitable for neonatal anatomy.

## Conclusion

This study supports the feasibility and safety of one stage definitive pull through for short segment Hirschsprung disease during the neonatal period when performed in an experienced high volume center, and suggests that long term bowel function is broadly comparable across single incision laparoscopic assisted, conventional laparoscopic assisted, and complete transanal approaches. Rather than implying a single superior technique, the findings emphasize that these approaches can be viewed as complementary options within a neonatal surgical pathway, with selection guided by operative goals such as the need for additional colonic mobilization, reliable identification of the transition zone, minimizing abdominal wall trauma, and optimizing cosmetic outcomes. Taken together, the results reinforce an individualized decision-making framework in which technique choice is aligned with patient specific anatomy and intraoperative assessment, while maintaining a consistent focus on safe execution, structured follow up, and functional recovery over time.

## Supporting information

S1 File STROBE checklist. (DOCX)

## Abbreviations

TERPT: Complete Transanal EndoRectal Pull-Through
SILEP: Single-Incision Laparoscopic-assisted Endorectal Pull-through
CLEP: Conventional Laparoscopic-assisted Endorectal Pull-through
HD: Hirschsprung’s Disease
HAEC: Hirschsprung-associated enterocolitis
MSS: Manchester Scar Scale
BFS: Rintala’s Bowel Function Score
STROBE: Strengthening the Reporting of Observational Studies in Epidemiology
