# Supplementary material for: Safety and feasibility of one-stage neonatal approach for short-segment Hirschsprung’s disease
Source: PLoS One. 2026 Jan 16;21(1):e0341212. doi: 10.1371/journal.pone.0341212 (PMC12810819; doi:10.1371/journal.pone.0341212)
Supplement: S1 File — (DOCX) [file pone.0341212.s001.docx]

# STROBE Statement Checklist for Cohort Study

**Manuscript title:** One Stage Neonatal Surgery for Hirschsprung’s Disease: Which Approach Offers the Best Long term Outcomes?
**Study design:** Retrospective cohort study

| Item / Section | Recommendation | Page No |
| --- | --- | --- |
| Title and abstract |  |  |
| 1a | Indicate the study design in the title or abstract. | 2 |
| 1b | Provide an informative, balanced summary of methods and main findings in the abstract. | 2 to 3 |
| Introduction |  |  |
| 2 | Explain the scientific background and rationale for the investigation. | 4 and 5 |
| 3 | State specific objectives and any prespecified hypotheses. | 5 |
| Methods |  |  |
| 4 | Present key elements of the study design early in the paper. | 2, 5 and 6 |
| 5 | Describe the setting, locations, and relevant dates including recruitment and follow up. | 5 and 6 |
| 6a | Give eligibility criteria and the sources and methods of selection of participants and describe follow up. | 6 and 7 |
| 6b | For matched studies give matching criteria and numbers of exposed and unexposed if applicable. | Not applicable |
| 7 | Clearly define all outcomes, exposures, predictors, potential confounders, and effect modifiers. | 7 and 8 |
| 8 | For each variable give data sources and details of assessment methods in each group. | 6 to 8 |
| 9 | Describe any efforts to address potential sources of bias. | 16 |
| 10 | Explain how the study size was determined or how the available sample was defined. | 6, 8 and 9 |
| 11 | Explain how quantitative variables were handled in the analyses including any groupings. | 8 |
| 12a | Describe all statistical methods including those used to control for confounding. | 8 |
| 12b | Describe any methods used to examine subgroups and interactions. | Not applicable |
| 12c | Explain how missing data were addressed. | 6 |
| 12d | If applicable explain how loss to follow up was addressed. | 10 and 12 |
| 12e | Describe any sensitivity analyses if performed. | Not applicable |
| Results |  |  |
| 13a | Report numbers of individuals at each stage of the study such as eligible, included, followed and analysed. | 6, 8 to 10 |
| 13b | Give reasons for non participation at each stage. | Not reported |
| 13c | Consider use of a flow diagram. | Not applicable |
| 14a | Give characteristics of study participants and information on exposures and potential confounders. | 8 to 10 |
| 14b | Indicate number of participants with missing data for each variable of interest. | 6 |
| 14c | Summarise follow up time for example mean, median, and total. | 9, 10 and 12 |
| 15 | Report numbers of outcome events or summary measures over time. | 10 and 11 |
| 16a | Give unadjusted and if applicable adjusted estimates with precision and specify confounders. | 8 to 11 |
| 16b | Report category boundaries when continuous variables were categorised. | 7, 10 and 11 |
| 16c | If relevant translate estimates of relative risk into absolute risk for a meaningful time period. | Not applicable |
| 17 | Report other analyses done such as subgroup analyses and interaction analyses. | 10 to 13 |
| Discussion |  |  |
| 18 | Summarise key results with reference to study objectives. | 11, 12 and 17 |
| 19 | Discuss study limitations considering potential sources of bias or imprecision and their direction and magnitude. | 16 |
| 20 | Give a cautious overall interpretation of results in light of objectives, limitations and other relevant evidence. | 11 to 17 |
| 21 | Discuss the generalisability of the study findings. | 16 and 17 |
| Other information |  |  |
| 22 | State the source of funding and the role of the funders for the present study. | 18 |
